# Paediatric Retinal Detachment in a Patient With Coexistent Stickler and Noonan Syndromes: The Importance of a Multidisciplinary Approach

**DOI:** 10.7759/cureus.101111

**Published:** 2026-01-08

**Authors:** Yasmin Bakr, Youssef Helmy, Catherine Qin, Dimitrios Kalogeropoulos, Marta Latasiewicz

**Affiliations:** 1 Ophthalmology, Stoke Mandeville Hospital, Buckinghamshire Healthcare NHS Trust, Aylesbury, GBR; 2 Ophthalmology, Kasr Al-Ainy Faculty of Medicine, Cairo University, Cairo, EGY

**Keywords:** genetic testing, noonan syndrome, rhegmatogenous retinal detachment, stickler syndrome, vitreoretinal disorders

## Abstract

We report the case of a 12-year-old boy of Zimbabwean descent with coexistent Stickler and Noonan syndromes who was referred to our unit after being diagnosed with a macula-on inferior rhegmatogenous retinal detachment (RRD). His ophthalmic history was otherwise unremarkable. Systemic assessment revealed dysmorphic features consistent with both syndromes, and genetic testing confirmed heterozygous pathogenic variants in *COL11A1* and *PTPN11*, inherited from the mother and father, respectively. The patient underwent multiple surgical interventions, including scleral buckling and pars plana vitrectomy with subretinal fluid drainage, laser retinopexy, and hexafluoroethane (C₂F₆) gas tamponade, ultimately achieving anatomical success and stable visual acuity at 11 months. To the best of our knowledge, this case is the first recorded coexistence of Stickler and Noonan syndromes in a patient with RRD, highlighting the significance of a multidisciplinary approach involving Ophthalmology, Genetics, and Paediatrics. Early detection of syndromic manifestation, comprehensive systemic evaluation, genetic diagnosis, and strategic surgical planning are essential to optimise functional and anatomical outcomes in paediatric patients with complex inherited vitreoretinal disorders.

## Introduction

Inherited vitreoretinal disorders constitute a significant cause of visual morbidity in the paediatric population, with Stickler syndrome (SS) being one of the most well-described clinical entities. SS, first described by Gunnar Stickler and colleagues as a hereditary arthro-ophthalmopathy [1], is commonly associated with rhegmatogenous retinal detachment (RRD). It is the most frequent clinical manifestation of type II and XI collagenopathies, which are a common cause of inherited RRD.

SS is related to six genes, defects in which result in an array of clinical features. Most cases are caused by deficiencies in type II collagen (*COL2A1*, chromosome 12) [2]. Less commonly, heterozygous pathogenic variants in *COL11A1* and *COL11A2* result in distinct Stickler subtypes. Deficiencies in type XI collagen, *COL11A1* (type 2 Stickler), *COL11A2* (type 3 Stickler), and *COL2A1*, also lead to SS [3]. Mutations in *COL11A1*, typically missense or splice-site alterations, may be associated with characteristic ophthalmic findings such as a type 2 "beaded" vitreous phenotype [4].

Clinically, *COL11A1*-related SS is often characterised by more pronounced craniofacial abnormalities, hearing loss, and type 2 vitreous anomalies, with a comparatively lower but still substantial lifetime risk of retinal detachment. Other associated features include deafness, cleft palate, Pierre-Robin sequence, joint hypermobility, and early-onset arthritis. Although the estimated risk of retinal detachment in *COL11A1*-related SS may be lower than that associated with *COL2A1* variants, it remains clinically significant and unpredictable [5]. Diagnosis can be challenging due to significant phenotypic variability, including within affected families.

While SS is a well-recognised condition that involves inherited vitreoretinal disorders, Noonan syndrome (NS) represents a distinct but clinically relevant genetic pathology that can also present with ocular abnormalities. NS is an autosomal dominant, genetically heterogeneous condition with age-dependent variable expressivity. It involves mutations in genes regulating the RAS-mitogen-activated protein kinase (RAS-MAPK) pathway, which governs cell proliferation and apoptosis. Over 50% of cases are associated with mutations in *PTPN11*. NS typically presents with short stature, congenital heart disease, facial dysmorphology, chest wall abnormalities, and cryptorchidism in males. Ophthalmic manifestations are common and include hypertelorism, ptosis, refractive errors, strabismus, amblyopia, and nystagmus, all of which necessitate comprehensive ophthalmic evaluation despite the lack of a strong documented association with RRD [6-8].

To date, no published literature has described an association between NS and retinal detachment, leaving its relevance in complex vitreoretinal presentations uncertain. Herein, we report the diagnostic and therapeutic approach of RRD in a paediatric case with an overlap of SS and NS. To the best of our knowledge, this is the first documented case with the coexistence of these two genetic abnormalities, highlighting the clinical and surgical challenges, as well as the crucial role of a multidisciplinary approach among Ophthalmology, Genetics, and Paediatrics.

## Case presentation

A 12-year-old boy of Zimbabwean descent was referred to our unit’s emergency eye clinic following the incidental detection of a macula-on inferior RRD in the right eye during a routine optician visit. He denied any prior visual symptoms, ocular trauma, or documented retinal pathology. His past ophthalmic history was otherwise unremarkable.

At initial review, the patient was using crutches while awaiting corrective surgery for a foot deformity. Although he had no formally documented systemic diagnoses at presentation, historical paediatric records noted speech delay, externally rotated hips, and elbow contractures. Clinical examination revealed several dysmorphic features consistent with NS, including hypertelorism, low-set ears, eyelid retraction, and down-slanting palpebral fissures. Features attributed predominantly to SS included midface hypoplasia, micrognathia, myopia, vitreous abnormalities, and fixed flexion deformities of the elbows, knees, and hips. Overlapping features such as micrognathia were noted but not assigned exclusively to either syndrome due to phenotypic overlap.

Family history revealed that the patient’s mother had high myopia (−7.00 dioptres) and multiple retinal detachments from the age of 37, but no recognised skeletal, craniofacial, or systemic features of SS. The maternal grandmother also had adult-onset RRDs, although the age of onset was not specified. The father had no known ocular or systemic abnormalities.

The patient’s complex ocular and systemic history prompted a multidisciplinary assessment by Paediatric Orthopaedics, Rheumatology, and Ophthalmology teams, and genetic testing was conducted. Genetic testing revealed that the patient was heterozygous for a likely pathogenic *COL11A1* variant (chromosome 1), inherited from his mother, confirming SS type 2. He was also heterozygous for a pathogenic *PTPN11* missense variant (chromosome 12), inherited from his father, confirming NS. Monoallelic pathogenic variants in *PTPN11* are known to cause autosomal dominant NS, with or without multiple lentigines.

**Figure 1:**
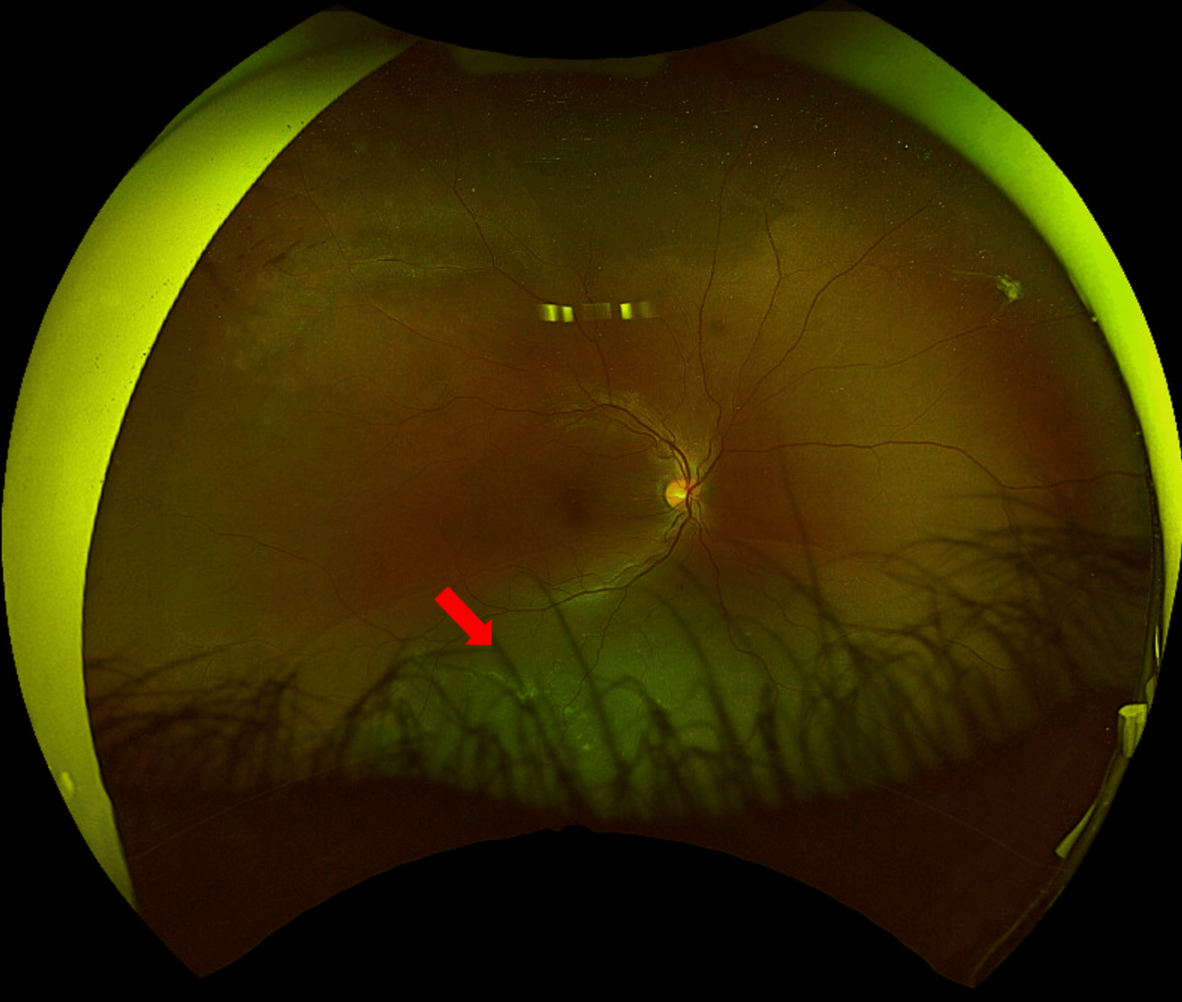
Optos wide-field photography of the right eye Right eye re-detachment (red arrow) after the first surgical approach. The re-detachment was treated with encircling buckle, drainage of subretinal through sclera and air.

His best corrected visual acuity (BCVA) was logMAR 0.10 (left eye) and 0.22 (right eye), with refraction of -3.00/-2.75 × 90° (right) and -1.75/-2.75 × 92° (left). Slit-lamp examination demonstrated deep anterior chambers with no evidence of inflammatory activity. The patient was bilaterally phakic, and the intraocular pressure was within normal limits, measuring 12 mmHg in both of his eyes. Dilated fundoscopy confirmed a right eye chronic inferior retinal detachment associated with a peripheral retinal hole (Figure 1).

**Figure 2:**
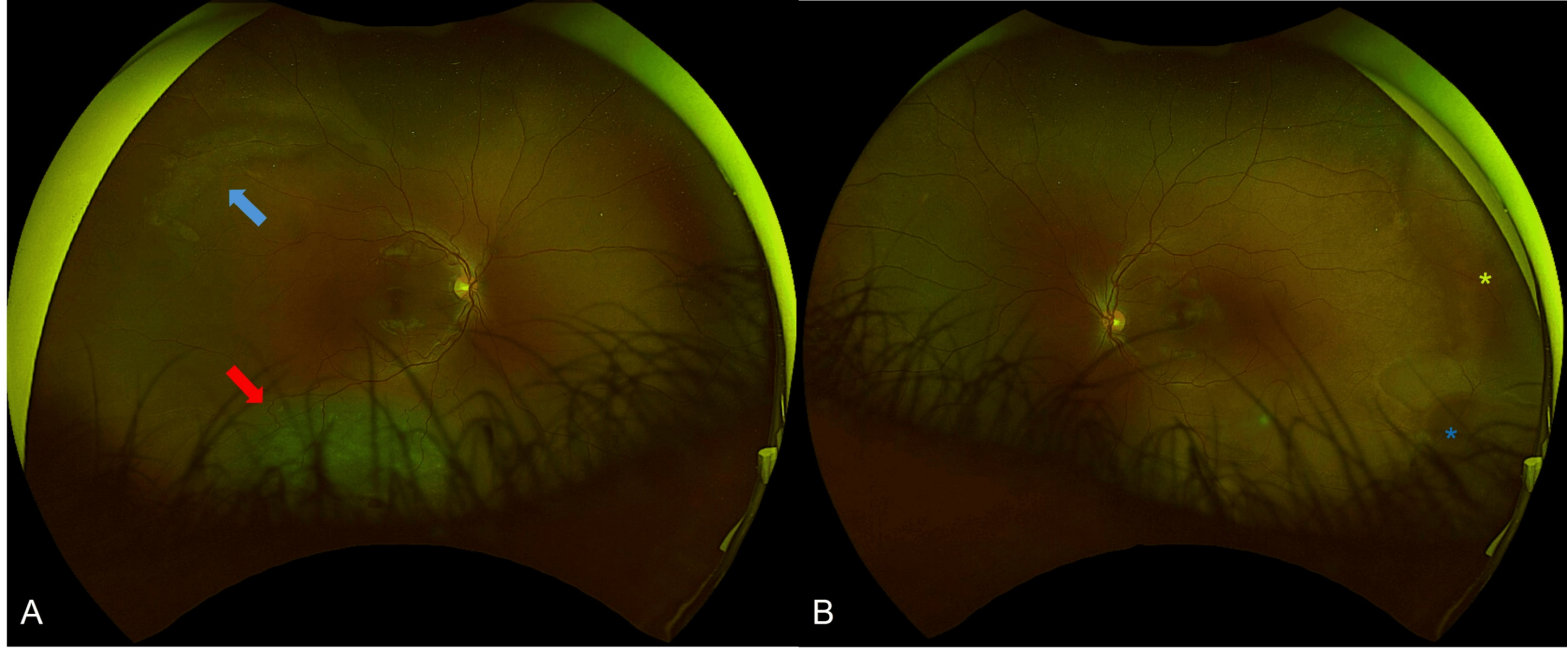
Optos wide-field photography of the right (A) and left (B) eyes. In the right eye there is an inferior retinal detachment (red arrow), as well as some lattice degeneration along the superotemporal arcade (blue arrow). In the left eye, white-without pressure can be observed at the temporal aspect (yellow asterisk), together with some possible schitic changes at the infero-temporal retina (blue asterisk). The detachment was treated with circumferential buckle, retinopexy, cryopexy and air.

**Figure 3:**
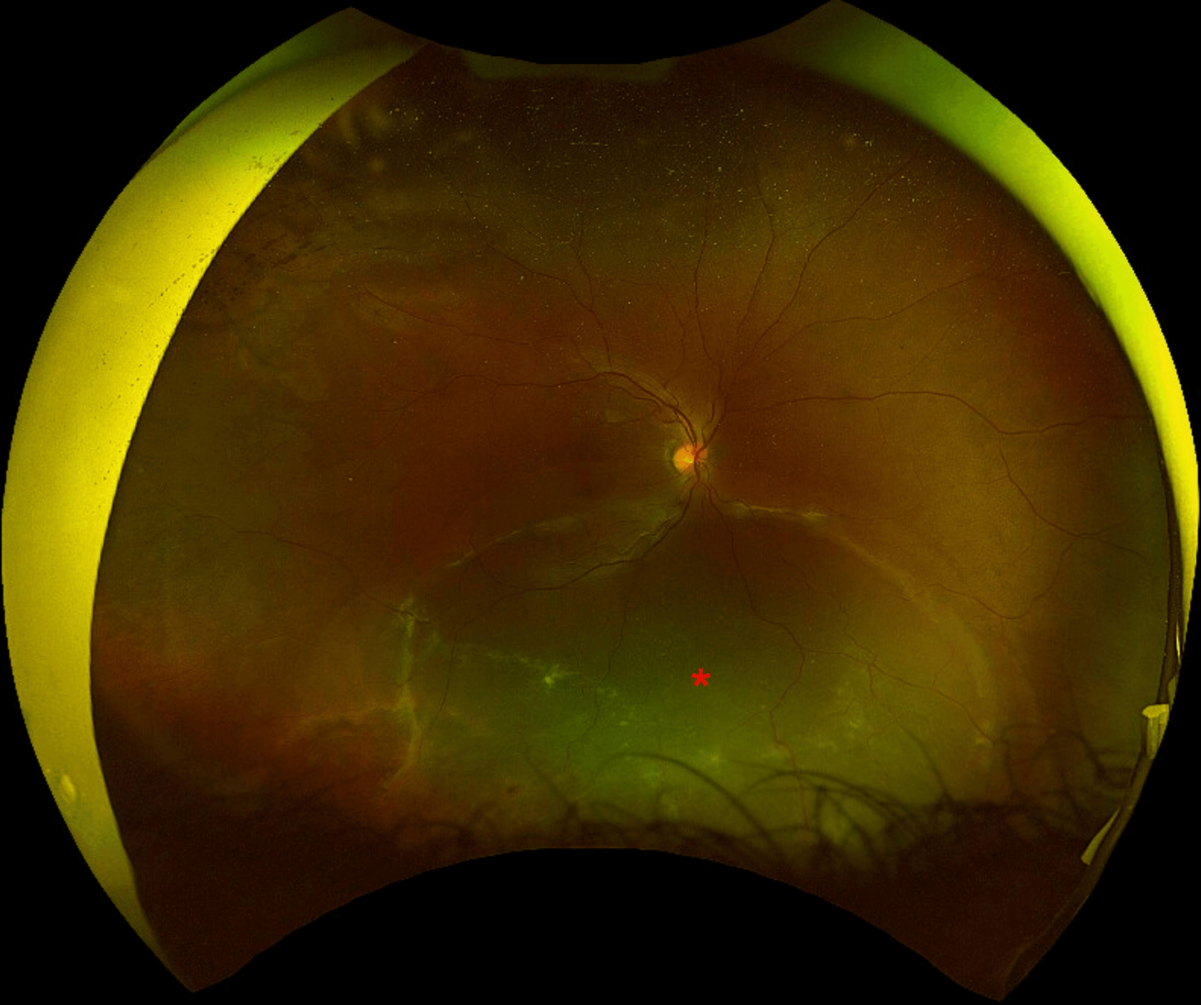
Optos wide-field photography of the right eye Right eye second macula-off re-detachment (red asterisk) that was managed with pars plana vitrectomy, 360^o^ prophylactic laser, endolaser around retinal tears and lattice, and C_2_F_6_.

Within a week, he underwent scleral buckling with cryopexy under general anaesthesia. Postoperatively, there was inadequate indentation at the break site, and the retina remained detached inferiorly with an attached macula. He underwent a second scleral buckle with drainage two months after the first. The second buckling procedure was also unsuccessful, with persistent inferior subretinal fluid and initially attached macula that later began to show subretinal fluid (Figures 2, 3).

**Figure 4:**
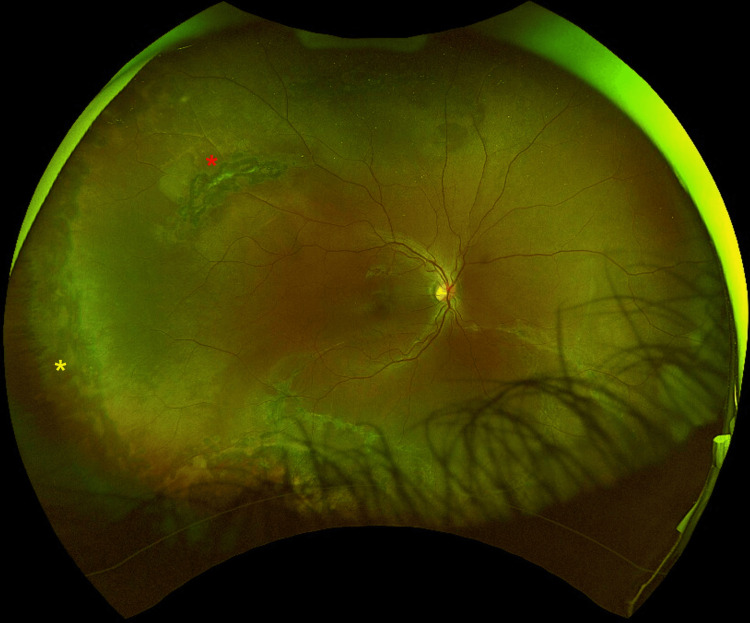
Fundal view with successful anatomical outcome after pars plana vitrectomy Retina is flat with no evidence of detachment or other acute pathology. Laser scars can be seen at the areas treated with 360^o^ laser (yellow asterisk) and around the lattice degeneration (red asterisk).

Given the repeated failures, the underlying syndromic vitreoretinopathy, and the progression towards macula-off detachment, it was decided to proceed with a third intervention, and more specifically with pars plana vitrectomy (PPV), subretinal fluid drainage, laser retinopexy, and 16% hexafluoroethane (C_2_F_6_) gas tamponade. This was performed three months after the second scleral buckle and led to anatomical success (Figure 4).

At the 11-month follow-up, the retina remained attached, and best-corrected visual acuity in the right eye was 0.20. The surgical procedures, key operative steps, timing, and visual outcomes are summarized in Table 1.

**Table 1:** Summary of surgical procedures and outcomes VA: visual acuity

Surgery	Date	Preoperative VA	Key steps	Postoperative outcome	Postoperative LogMAR VA
1. Buckle	September 2023	0.10	Indirect ophthalmoscope cryo-retinopexy, #277 circumferential segment, no drainage	Inferior persistent retinal detachment macula-on	0.20
2. Buckle	November 2023	0.20	Candelier-asssisted removal of #277 segment cryo-retinopexy #40 encircling band, drainage, and air	Inferior persistent retinal detachment, Initially macula-on, macula-off (February 2024)	0.50
3. Vitrectomy	February 2024	0.50	25-gauge pars plana vitrectomy, PVD-induced drainage of fluid through break 360^o^ laser retinopexy C2F6 gas tamponade	Flat retina	0.20

## Discussion

This case highlights the importance of a multidisciplinary approach in the evaluation and management of paediatric RRD with associated systemic features. The coexistence of SS and NS presents unique diagnostic and surgical challenges due to overlapping phenotypic features and complex vitreoretinal anatomy.

Idiopathic RRD in children should always prompt consideration of inherited causes such as SS. RRDs associated with SS are particularly challenging to treat due to difficulties in paediatric examination, often late presentation, as multiple factors increase the complexity of repair. These include multiple retinal tears, giant retinal tears, pathological vitreous, and proliferative vitreoretinopathy (PVR). A study by Reddy et al. reported that the average number of RRD repair surgeries required in paediatric patients was 3.1, with limited improvement in postoperative visual acuity [9].

In our patient, repeated scleral buckling failure was likely attributable to syndromic vitreoretinopathy, difficulty in achieving adequate buckle support at the break site, and altered vitreoretinal adhesion, factors commonly encountered in SS. This ultimately necessitated conversion to vitrectomy to achieve anatomical success.

Establishing an early genetic diagnosis is pivotal, as prophylactic treatment, such as peripheral 360° retinopexy of the fellow eye, can prevent retinal detachments in certain variants of the disease. The Cambridge prophylactic cryotherapy protocol, developed for type 1 SS, demonstrated that patients who did not receive prophylactic cryotherapy had a 7.4-fold higher risk of retinal detachment compared with those who received treatment [10]. While evidence for prophylaxis in *COL11A1*-related SS is limited, awareness of genetic subtype remains crucial for surveillance and counselling.

Genotype-phenotype correlations in NS are well-described. The *PTPN11* gene is commonly associated with typical facial features, pulmonary valve stenosis, easy bruising, and cryptorchidism. Although our patient had facial markers like hypertelorism and down-slanting palpebral fissures, he lacked other phenotypic features typically linked to this mutation. The variable expression and incomplete penetrance of NS highlight the importance of early referral to paediatrics or clinical genetics when clinical suspicion arises. It is also imperative that patients with suspected NS undergo a thorough ophthalmological assessment due to several amblyogenic factors associated with the condition, including but not limited to ptosis, strabismus, and astigmatism [6-8].

Based on this case, we propose several considerations. First, pediatric rhegmatogenous RRD in the presence of systemic features should prompt early multidisciplinary evaluation. Second, establishing a genetic diagnosis is crucial to guide surveillance and prophylactic strategies. Third, surgical management may require multiple interventions due to abnormal vitreoretinal anatomy. Finally, a comprehensive ophthalmic examination is essential in patients with SS and/or NS, even in the absence of overt systemic features.

## Conclusions

To our knowledge, there are no previously published reports describing retinal detachment in a patient with coexistent SS and NS. This overlap has important clinical implications, including increased surgical complexity, diagnostic uncertainty, and the need for coordinated care across specialties. This case highlights the challenges and significance of a coordinated, multidisciplinary approach to paediatric RRD in individuals with overlapping syndromes, providing both surgical insight and guidance for systemic evaluation.
